# Cellular response to 5-fluorouracil (5-FU) in 5-FU-resistant colon cancer cell lines during treatment and recovery

**DOI:** 10.1186/1476-4598-5-20

**Published:** 2006-05-18

**Authors:** Paula M De Angelis, Debbie H Svendsrud, Katherine L Kravik, Trond Stokke

**Affiliations:** 1 Institute of Pathology, Section for Molecular Chemoresistance, Rikshospitalet-Radiumhospitalet HF, Oslo, Norway; 2 Institute for Cancer Research, Rikshospitalet-Radiumhospitalet HF, Oslo, Norway

## Abstract

**Background:**

Treatment of cells with the anti-cancer drug 5-fluorouracil (5-FU) causes DNA damage, which in turn affects cell proliferation and survival. Two stable wild-type *TP53 *5-FU-resistant cell lines, ContinB and ContinD, generated from the HCT116 colon cancer cell line, demonstrate moderate and strong resistance to 5-FU, respectively, markedly-reduced levels of 5-FU-induced apoptosis, and alterations in expression levels of a number of key cell cycle- and apoptosis-regulatory genes as a result of resistance development. The aim of the present study was to determine potential differential responses to 8 and 24-hour 5-FU treatment in these resistant cell lines. We assessed levels of 5-FU uptake into DNA, cell cycle effects and apoptosis induction throughout treatment and recovery periods for each cell line, and alterations in expression levels of DNA damage response-, cell cycle- and apoptosis-regulatory genes in response to short-term drug exposure.

**Results:**

5-FU treatment for 24 hours resulted in S phase arrests, p53 accumulation, up-regulation of p53-target genes on DNA damage response (*ATF3*, *GADD34*, *GADD45A*, *PCNA*), cell cycle-regulatory (*CDKN1A*), and apoptosis-regulatory pathways (*FAS*), and apoptosis induction in the parental and resistant cell lines. Levels of 5-FU incorporation into DNA were similar for the cell lines. The pattern of cell cycle progression during recovery demonstrated consistently that the 5-FU-resistant cell lines had the smallest S phase fractions and the largest G_2_(/M) fractions. The strongly 5-FU-resistant ContinD cell line had the smallest S phase arrests, the lowest CDKN1A levels, and the lowest levels of 5-FU-induced apoptosis throughout the treatment and recovery periods, and the fastest recovery of exponential growth (10 days) compared to the other two cell lines. The moderately 5-FU-resistant ContinB cell line had comparatively lower apoptotic levels than the parental cells during treatment and recovery periods and a recovery time of 22 days. Mitotic activity ceased in response to drug treatment for all cell lines, consistent with down-regulation of mitosis-regulatory genes. Differential expression in response to 5-FU treatment was demonstrated for genes involved in regulation of nucleotide binding/metabolism (*ATAD2*, *GNL2*, *GNL3*, *MATR3*), amino acid metabolism (*AHCY*, *GSS*, *IVD*, *OAT*), cytoskeleton organization (*KRT7*, *KRT8*, *KRT19*, *MAST1*), transport (*MTCH1*, *NCBP1*, *SNAPAP*, *VPS52*), and oxygen metabolism (*COX5A*, *COX7C*).

**Conclusion:**

Our gene expression data suggest that altered regulation of nucleotide metabolism, amino acid metabolism, cytoskeleton organization, transport, and oxygen metabolism may underlie the differential resistance to 5-FU seen in these cell lines. The contributory roles to 5-FU resistance of some of the affected genes on these pathways will be assessed in future studies.

## Background

5-fluorouracil is a chemotherapeutic drug used worldwide in the treatment of metastatic colorectal cancer, either alone or in combination with irinotecan, a topoisomerase I inhibitor. 5-FU is considered to be purely an S phase-active chemotherapeutic agent, with no activity when cells are in G_0 _or G_1 _[1]. It is well-established that treatment of cells with 5-FU causes DNA damage, specifically double-strand (and single-strand) breaks, during S phase due to the misincorporation of FdUTP into DNA [2,3]. However, damage to DNA can occur in all cell cycle phases in proliferating cells, and the repair mechanisms involved vary in the different phases of the cell cycle [4,5]. DNA damage checkpoint pathways in G_1_, S, and G_2 _couple DNA damage detection to inhibition of cell cycle progression, activation of DNA repair, maintenance of genomic stability, and when damage is beyond repair, to initiation of cellular senescence [6].

The position of tumor cells in the cell cycle and the ability to undergo apoptosis in response to drug treatment together play an important role in the sensitivity of tumor cells to chemotherapy. 5-FU has a complicated mechanism of action with several enzymes involved in its metabolic activation [7]. It inhibits thymidylate synthase as its main mechanism of action, leading to depletion of dTTP. Overexpression of thymidylate synthase has been shown to be associated with 5-FU resistance in colorectal cancer [8,9], but it is also likely that other alterations, for example, to crucial genes on cell cycle and apoptotic regulatory pathways, underlie the development of resistance. Two independent 5-FU-resistant cell lines, designated ContinB and ContinD, were recently generated from parental HCT116 colon cancer cells via continuous exposure to 5-FU, and characterized for genotypes, phenotypes, and gene expression associated with the generation of 5-FU resistance [10]. Compared to parental HCT116 cells, the resistant cell lines demonstrated moderate (ContinB) to strong (ContinD) resistance to 5-FU and up-regulation of *TYMS*. Cellular phenotypes such as reduced apoptosis and more aggressive growth relative to the parental HCT116 cell line characterized both resistant cell lines. This was consistent with up-regulation of apoptosis-inhibitory genes (*IRAK1, MALT1, BIRC5*), positive growth-regulatory genes (*CCND3, CCNE2*), DNA repair genes (*FEN1, FANCG*), and metastasis signature genes (*LMNB1, F3 TMSNB*), and down-regulation apoptosis-promoting genes (*BNIP3*, *BNIP3L*, *FOXO3A*) and negative growth-regulatory genes (*AREG*, *CDKN1A*, *CCNG2*, *GADD45A*) in one or both resistant cell lines. Both 5-FU-resistant cell lines retained the wild-type *TP53 *genotype characteristic of the parental HCT116 cells [10]. In the present work, the cellular responses of HCT116 parental and 5-FU-resistant cell lines to short-term 5-FU treatment were characterized and compared. Given the fact that the 5-FU-resistant cell lines displayed reduced apoptosis and more aggressive growth phenotypes compared to the parental cells as a consequence of resistance development, it was of interest to determine potential differential responses to 5-FU during short-term 5-FU challenge. We investigated cell cycle effects and apoptosis induction throughout treatment and recovery periods for each cell line, as well as changes in expression levels of DNA damage response-, cell cycle- and apoptosis-regulatory genes (among others) that occur within the first 24 hours in response to 5-FU treatment. Characterizations of the cellular responses to short-term drug treatment in resistant colorectal cancer cells will facilitate a better understanding of the multiple mechanisms involved in drug response and development of 5-FU resistance.

## Results

### Cell proliferation, cell cycle distribution, and apoptosis during recovery from 5-FU treatment

**Figure 1:**
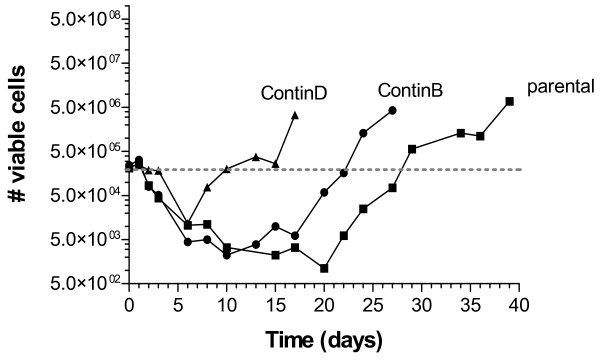
**Cell counts during recovery periods following drug removal**: cell counts were measured throughout the respective recovery periods for each cell line following a shift to drug-free medium at 24 hours (Day 1). The dashed (---) line shows the number of viable cells in untreated exponentially-growing cultures.

Cells were treated with 5-FU for 24 hours and followed for up to 40 days in drug-free medium to determine cell counts, cell cycle distributions, and apoptotic fractions (Figure 1). Cell counts decreased during the early period of recovery following drug removal (up to and including day 6) for all cell lines, after which point they flattened out for the parental and ContinB cells (Figure 1). After day 7, the cell counts for ContinD increased steadily. The cell counts for ContinB and parental cultures began to increase after about 15 and 20 days, respectively.

**Figure 2:**
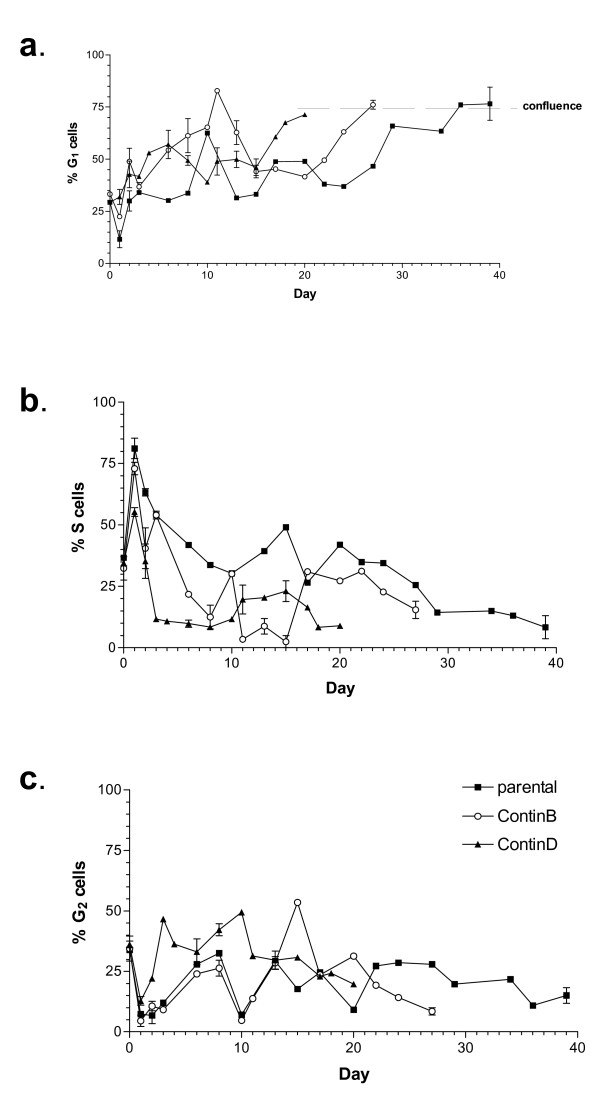
**Cell cycle distributions during recovery periods following drug removal**: fractions of cells in (a)G_1_, (b)S, and (c)G_2_ cell cycle phases were measured at intervals during the   respective recovery periods for each cell line.

Cell cycle analyses were performed to elucidate the patterns and timeframes of cell cycle progression during recovery in each 5-FU-treated cell line (Figure 2). After an initial accumulation in S phase during the first 24 hours with drug treatment (see later), the S phase fractions decreased in all cell lines during the early period of recovery following drug removal, concomitant with increases in the G_1 _and G_2_/M fractions. Overall, the 5-FU-resistant cell lines had the smallest S phase fractions (Figure 2b), and in the case of the ContinD cell line, the largest G_2_/M fractions (Figure 2c). The S phase fractions increased again at 8 and 15 days for ContinD and ContinB cells, respectively. The cell cultures were eventually allowed to reach full confluence, evidenced by an increase in the G_1 _fraction and decreases in the S and G_2_/M phase fractions in all the cell lines.

**Figure 3:**
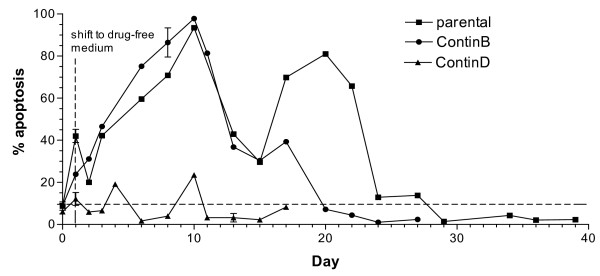
**Apoptotic fractions during recovery periods following drug removal**: apoptosis induction in 5-FU-treated HCT116 cell lines during the respective recovery periods for each cell line. The dashed line shows the levels of natural (spontaneous) apoptosis in untreated control cells.

5-FU-induced DNA damage resulted in large differences in apoptosis induction in the HCT116 cell lines during treatment and recovery (Figure 3), with the highest levels of apoptosis observed in the parental cells and the lowest in the ContinD cells. Following removal of 5-FU at 24 hours, apoptosis levels increased in the parental and ContinB cells, until they peaked at over 80% on day 10, after which they decreased to 30%. On day 15 the apoptotic fractions began to increase again, but only for the parental cells, peaking at about 80% on day 20, and then the levels gradually decreased to control levels at day 24. There was no 5-FU induced increase in the fraction of apoptotic ContinD cells (compared to the levels of spontaneous apoptosis in the untreated cells).

### 5-FU incorporation and cell cycle progression during the initial 24 hour treatment period

**Figure 4:**
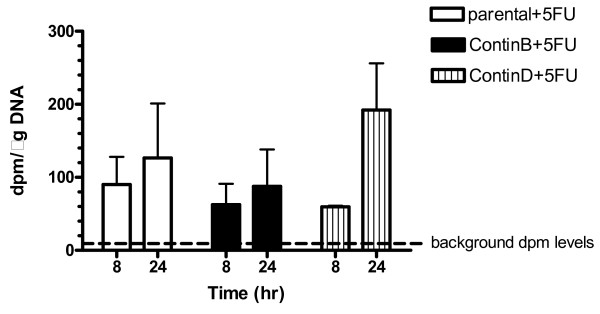
**Incorporation of 5-FU into DNA of parental and 5-FU-resistant HCT116 cell lines in response to 8 and 24-hr. 5-FU treatment**: Incorporation of 5-FU into DNA is given as the ratio of disintegrations per minute (dpm) per μg DNA. The dashed line represents background dpm levels.

The large differences in cell growth and apoptosis during recovery suggested that there might be differential responses to 5-FU in the cell lines during the first 24 hours of treatment. The reduced apoptosis of ContinB/D cells to 5-FU compared to the parental cells could have been due to decreased incorporation of 5-FU into DNA. At 8 hours, the parental cell line incorporated more [6-^3^H]5-FU into DNA than did either of the resistant cell lines, but the differences were not significant (Figure 4). At 24 hours, the ContinD cell line showed the highest levels of 5-FU incorporation into DNA, whereas the ContinB cell line had the lowest levels (p < 0.05). However, neither of the two resistant cell lines showed significant differences in incorporation relative to the parental cells.

**Figure 5:**
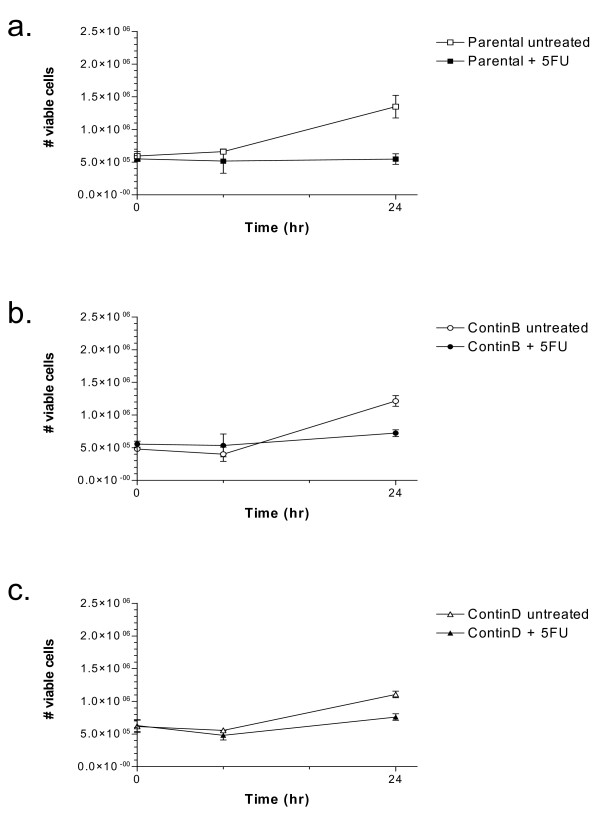
**Cell growth in parental and 5-FU-resistant HCT116 cell lines in response to 5-FU treatment for 24 hours**: cell growth at 8 and 24 hours after addition of 5-FU to the media in (a) parental cells, (b) ContinB cells, (c) ContinD cells.

**Figure 6:**
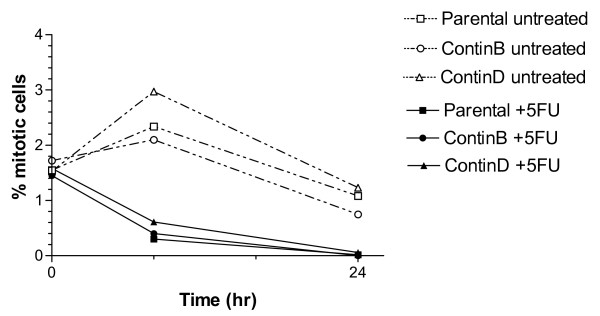
**Mitotic fractions in parental and 5-FU-resistant HCT116 cell lines in response to 5-FU treatment for 24 hours**: mitotic fractions in all cell lines at 8 and 24 hours after 5-FU addition, showing gradual cessation of mitosis over the 24-hour treatment period.

**Figure 7:**
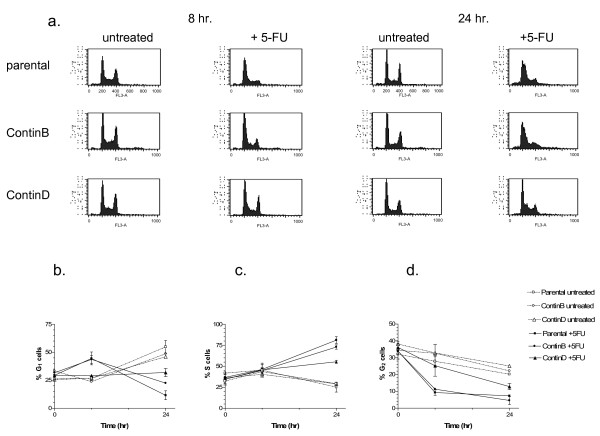
**Cell cycle progression in parental and 5-FU-resistant HCT116 cell lines in response to 5-FU treatment for 24 hours**: (a) cell cycle distributions showing G_1_/S boundary arrests at 8 hours and S phase arrests at 24 hours, showing S phase arrests at 24 hours; (b)-(d) G_1_, S and G_2_M fractions in all cell lines at 8 and 24 hours after 5-FU addition.

We further investigated whether there were differences in growth or cell cycle progression during the first 24 hours of 5-FU treatment. The growth of HCT116 parental cells was completely inhibited at 24 hours (Figure 5). The cell number increased after 24 hours of 5-FU treatment for the ContinD and to a smaller degree for the ContinB cell lines, but less than in the corresponding controls. However, since the fraction of apoptotic cells was increased at 24 hours for the parental (and to a smaller degree ContinB cells; Figure 3), some cells in these cultures may still have divided during the 24 hour period, in agreement with the non-zero mitotic fractions observed at 8 hours (Figure 6). No mitotic cells were observed at 24 hours. The distribution of cells in the G_1_, S, and G_2_(/M) phases of the cell cycle was measured by staining for DNA content (Figure 7). A G_1_(/S) arrest occurred in the parental and ContinB cells at 8 hours after 5-FU addition, evidenced by a larger fraction of cells in the G_1 _phase. At 8 hours, the size of the G_1 _fraction in 5-FU-treated ContinD cells was similar to that measured for its untreated control. S phase fractions in all 5-FU-treated cell lines were equivalent in size and similar to those measured in the respective untreated controls at the 8 hour timepoint. The sizes of the G_2_(/M) fractions in 5-FU-treated parental and ContinB cell lines were smaller than their respective controls at 8 hours, but in ContinD cells the size of the G_2 _fraction was similar to that measured for the untreated controls. At 24 hours, the G_1 _fractions in all 5-FU-treated cell lines were smaller and the S phase fractions were larger compared to their respective untreated controls, indicating release of the arrested cells at the G_1_/S boundary and movement into S phase. The cell cycle histograms show directly synchronized populations of cells in S phase caused initially by the G_1_/S arrest and subsequent release of these cells into S phase (Figure 7). Parental HCT116 cells had the largest S phase accumulation (80% S phase cells), whereas ContinB and ContinD cells had smaller S phase accumulations (70% and 52%, respectively) compared to 25% in the respective untreated controls (Figure 7c). The G_2 _fractions in the 5-FU-treated cells at 24 hours were smaller relative to those measured for untreated control cells, probably reflecting the slow movement of cells through S phase. ContinD cells had the largest G_2 _fraction compared to the other 5-FU-treated cell lines.

### Expression of DNA damage response, cell-cycle regulatory, and apoptosis-regulatory genes

**Figure 8:**
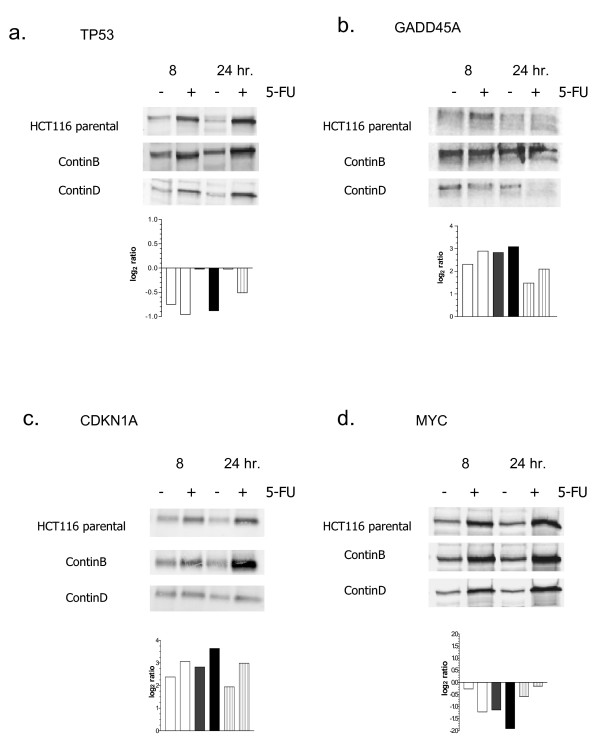
**DNA damage response and cell cycle-regulatory protein and transcript levels in 5-FU-treated parental and resistant HCT116 cell lines**: (a) p53 protein and TP53 transcript levels at 8 and 24 hours in 5-FU-treated HCT116 cell lines and their corresponding untreated controls; (b) GADD45A protein and transcript levels at 8 and 24 hours in 5-FU-treated HCT116 cell lines and their corresponding untreated controls; (c) CDKN1A protein and transcript levels at 8 and 24 hours in 5-FU-treated HCT116 cell lines and corresponding untreated controls; (d) MYC protein and transcript levels at 8 and 24 hours in 5-FU-treated HCT116 cell lines and corresponding untreated controls. For the bar charts that present gene expression levels, colorless bars depict the parental cell line, black bars the ContinB cell line, and vertically-striped bars the ContinD cell line.

Since neither the incorporation of 5-FU nor differences in cell cycle arrest could explain the large differences in 5-FU resistance and induction of apoptosis, we investigated the gene expression patterns of the cell lines in response to 5-FU challenge. Table 1 summarizes the microarray gene expression data for altered genes localized to DNA damage response, cell cycle-regulatory and apoptosis-regulatory pathways. The alterations in gene expression levels are in response to 5-FU treatment, but information about whether these genes were altered as a consequence of resistance development [10] are also included. For some genes, protein levels were measured in addition to gene transcript levels at 8 and 24 hours (Figure 8). At 8 hours, p53 protein levels were 2.0, 1.8, and 1.4 fold higher in 5-FU-treated parental, ContinB and ContinD cells respectively relative to their respective untreated controls (Figure 8a). At 24 hours, p53 protein levels had increased further relative to control levels; levels were 2.3, 3.0, and 2.1-fold higher in 5-FU-treated parental, ContinB and ContinD cells respectively. A number of important genes located on DNA damage response pathways were scored as altered in the 5-FU-treated cells relative to the untreated control cells following exposure to 5-FU. The *ATF3*, *GADD34*, *GADD45A*, *PCNA*, and *TP53I3 *genes were all up-regulated at 8 and/or 24 hours in response to 5-FU treatment in all HCT116 cell lines relative to untreated controls. *GADD45A *transcript levels were highest in 5-FU-treated ContinB cells at 24 hours, nearly 10-fold higher than in the untreated control. (*GADD45A *expression levels measured by real-time RT-PCR correlated well with those measured using the 13 K microarrays (r = 0.83, p < 0.05)). However, GADD45A protein levels increased only 10% in the treated parental and ContinB cell lines at 8 hours, and in ContinD cells they had actually decreased about 10%; at 24 hours there was no change in GADD45A protein levels in the treated parental cells, whereas we measured a decrease of 1.3 and 1.8 fold respectively for treated ContinB and ContinD relative to their respective controls (Figure 8b). *RAD23A *and *XPC *were down-regulated and up-regulated respectively in ContinB cells at 8 hours, but not in the other cell lines at any timepoint. *MSH2 *was down-regulated in the 5-FU-treated parental and ContinB cell lines.

**Table 1:** Differential gene expression in parental HCT116 and 5-FU-resistant cell lines: DNA damage response/repair, cell cycle, and apoptosis regulatory pathways.

Gene symbol	parent al 8 hr 5 FU^a^	parenta l 24 hr 5 FU ^a^	Contin B 8 hr 5 FU ^a^	Contin B 24 hr 5 FU ^a^	Contin D 8 hr 5 FU ^a^	Contin D 24 hr 5 FU ^a^	altered in ContinB as a result of resistance development ^b^	altered in ContinD as a result of resistance development ^b^	Regulatory pathway/function
									***DNA damage response/DNA repair***
*ATF3*	**2.1**	**3.1**	**1.5**	**2.3**	**1.8**	**2.8**	-0.7	**-1.2**	protective response of human cells to ionizing radiation
*GADD34*	0.9	**1.4**	0.9	**1.3**	0.8	**1.5**	-0.1	-0.2	cellular response to stress and DNA damage stressful growth arrest conditions, treatment with DNA-damaging
*GADD45A*	**2.3**	**2.9**	**2.8**	**3.1**	**1.5**	**2.1**	**-1.1**	-0.3	agents
*MMS2*	0.3	0.7	0.2	0.6	-0.1	-0.1	na	na	cellular response to stress; regulation of DNA repair
*MSH2*	**-1.0**	**-1.1**	**-1.2**	-0.7	-0.2	-0.8	-0.3	0.8	mismatch repair
*PCNA*	**1.1**	**2.6**	0.9	**2.2**	0.6	**1.3**	0.8	0.8	involved in DNA replication and repair
*RAD23A*	-0.2	-0.4	**-1.6**	-0.5	-0.1	0.1	na	na	nucleotide excision repair
*TP53*	-0.8	**-1.0**	0.0	-0.9	0.0	-0.5	0.5	0.4	DNA damage response, negative regulator of cell growth
*TP53I3*	-0.7	**2.3**	0.7	**2.5**	0.4	**2.5**	na	na	cellular response to oxidative stresses and irradiation
*XPC*	0.0	0.4	**1.3**	0.8	0.2	0.5	-0.6	-0.4	nucleotide excision repair
									
									***Cell cycle/cell proliferation***
*AREG*	0.2	**1.9**	0.8	**3.1**	-0.1	**1.8**	**-2.2**	**-2.1**	positive regulator of cell growth; inhibits apoptosis together with IGF1
*BUB1*	**-1.3**	**-1.1**	-0.6	-0.3	-0.5	-0.9	0.4	0.6	spindle checkpoint function
*BUB1B*	**-1.4**	**-2.5**	-0.9	**-1.8**	-0.9	**-1.4**	0.6	0.6	spindle checkpoint function
*CCNB1*	-0.3	-0.5	-0.5	-0.4	-0.5	**-1.8**	na	na	regulates G_2_M cell cycle transition
*CCNB2*	-0.7	**-1.1**	-0.7	-0.7	-0.4	**-1.6**	0.0	0.5	regulates G_2_M cell cycle transition
*CCNC*	**-1.0**	**-1.9**	**-2.1**	**-2.0**	-0.4	-0.5	-0.4	-0.3	involved in G_1 _cell cycle regulation
*CCND3*	0.7	**1.6**	0.9	**2.4**	0.4	**1.0**	**1.7**	0.1	regulates G_1_/S cell cycle transition
*CCNG1*	**-1.8**	**-1.1**	-0.4	**-1.3**	-0.3	-0.5	**-1.4**	-0.3	growth inhibitory activity linked to ARF-p53 and pRb pathways.
*CDC25B*	**-1.6**	**-1.4**	-0.9	**-1.2**	-0.3	-0.6	0.2	-0.7	dephosphorylates CDC2 to allow progression to mitosis
*CDC6*	0.7	**1.1**	-0.6	0.0	0.2	0.2	0.8	**1.0**	regulator at early steps of DNA replication
*CDCA5*	0.2	**1.4**	-0.1	0.5	0.2	0.2	na	na	G_1_/S transition of cell cycle
*CDKN1A*	**2.4**	**3.1**	**2.8**	**3.6**	**1.9**	**3.0**	-0.5	**-1.1**	negative regulator of cell cycle progression at G1
*CHC1*	**-1.5**	-0.2	**-1.4**	**-1.1**	-0.4	-0.2	**1.8**	0.7	regulator of chromosome condensation
*GTF2B*	-0.7	-0.4	**-1.1**	**-1.3**	-0.2	0.2	-0.8	-0.1	regulation of transcription, DNA-dependent
*H3F3B*	-0.7	-0.6	**-1.9**	**-1.1**	-0.1	0.0	-0.1	-0.1	chromosome organization and biogenesis; nucleosome assembly
*IRF6*	-0.3	0.5	-0.6	0.1	0.0	**1.2**	na	na	regulation of transcription, DNA-dependent
*LIPH*	0.2	**-1.2**	-0.5	-0.4	0.0	0.0	na	na	stimulation of cell proliferation and motility
*MCM3*	-0.6	**-1.5**	-0.4	**-2.1**	0.0	**-1.9**	**1.0**	0.8	initiation of genome replication; formation of replication forks
*MKI67*	-0.8	**-1.2**	0.0	-0.4	-0.1	-0.4	0.8	0.9	antigen identified by monoclonal antibody Ki-67; cell proliferation
*MYC*	-0.3	**-1.2**	**-1.1**	**-1.9**	-0.6	-0.2	-0.3	-0.5	dual signaling for cell growth and cell death
*NAP1L1*	-0.3	-0.8	-0.9	**-1.9**	-0.4	-0.7	na	na	DNA replication; positive regulation of cell proliferation
*NBL1*	**-1.1**	**-1.3**	-0.7	-0.5	0.0	-0.1	na	na	transcription factor; negative regulator of cell cycle
*NDEL1*	-0.6	-0.3	**-1.5**	**-1.1**	-0.3	0.0	0.3	-0.1	thiol-activated peptidase phosphorylated in M phase of the cell cycle
*NEK1*	-0.7	**-1.2**	-0.2	0.0	-0.1	-0.5	na	na	DNA damage response pathway at the G2/M transition
*NEK4*	**-1.0**	**-1.2**	-0.6	-0.8	-0.5	**-1.2**	na	na	involved in mitosis
*NEK9*	**-1.2**	**-1.1**	nd	-0.1	-0.2	-0.4	na	na	binds Ran GTPase and regulates mitotic progression
*PARD3*	-0.8	0.3	-0.6	**-1.5**	-0.4	-0.1	nd	nd	asymmetric cell division; establishment/maintenance of cell polarity
*PDAP1*	0.2	**-1.3**	0.5	-0.3	0.2	-0.5	0.9	0.8	cell proliferation; signal transduction
*PDXP*	-0.3	**-1.3**	0.4	-0.3	0.1	-0.5	na	na	maintaining biochemical homeostasis required for proper spindle assembly/function; regulates G2M
*PLK*	**-2.1**	**-2.3**	**-1.8**	**-2.8**	-0.5	**-1.6**	**1.5**	0.7	progression
*PPP2CB*	0.8	**1.7**	**1.0**	**2.2**	0.2	**1.1**	0.1	0.0	negative regulator of cell growth and division
*PVT1*	**-1.4**	-0.7	0.6	0.4	0.1	0.4	na	na	MYC activator
*RAN*	-0.6	-0.8	**-1.2**	**-1.6**	0.0	-0.3	0.0	0.2	translocation of RNA and proteins through nuclear pore complex
*RARRES2*	**-1.6**	-0.9	-0.1	-0.6	-0.1	-0.1	nd	nd	growth inhibitory and cell differentiation activities
*RFP*	-0.6	-0.4	**-1.0**	**-1.1**	-0.1	0.2	na	na	cell proliferation; regulation of transcription, DNA-dependent
*RNF4*	-0.2	**-1.1**	0.1	-0.4	-0.1	0.1	0.3	0.2	transcription regulator; inhibits activity of TRPS1
*RRM2*	0.9	**1.4**	0.9	**1.0**	0.4	0.1	0.4	0.8	catalyzes formation of deoxyribonucleotides from ribonucleotides
*RUNX3*	-0.7	-0.8	-0.6	**-1.3**	-0.1	0.0	0.1	-0.3	cell proliferation; regulation of transcription, DNA-dependent
*STK6*	**-2.1**	**-1.5**	**-1.1**	**-1.4**	-0.8	**-2.1**	**1.0**	0.8	microtubule formation at spindle pole during chromosome segregation
*TIMP1*	0.1	-0.7	0.4	-0.3	-0.3	**-1.4**	-0.5	-0.5	positive regulation of cell proliferation
*TOP1*	-0.9	**-1.4**	**-1.8**	-0.1	-0.4	-0.1	-0.4	-0.4	control of DNA topology during transcription
*YWHAE*	0.4	**-1.2**	0.4	-0.2	-0.1	-0.3	**-1.2**	-0.6	member of 14-3-3 family of proteins which mediate signal transduction
									
									***Apoptosis***
*AVEN*	**0.2**	**1.5**	**1.0**	**1.8**	0.1	**1.0**	0.8	-0.2	inhibits Apaf-1-mediated caspase activation
*BAK1*	-0.5	-0.8	-0.2	-0.6	-0.2	-0.1	0.7	-0.2	apoptosis promoter
*BAX*	na	na	na	na	na	na	0.5	-0.3	apoptosis promoter
*BCL2*	nd	nd	nd	nd	nd	nd	nd	nd	apoptosis inhibitor
*BIRC5*	-0.2	**-1.2**	nd	0.0	-0.1	-0.3	**1.2**	0.7	apoptosis inhibitor
*BNIP3*	na	na	na	na	na	na	**-1.0**	-0.1	apoptosis promoter
*BNIP3L*	**-1.3**	**-2.0**	nd	**-2.0**	**-1.4**	**-1.0**	**-2.7**	-0.5	apoptosis promoter
*CASP3*	0.0	-0.5	**-1.7**	**-1.2**	-0.3	-0.1	0.0	0.1	apoptosis promoter
*FAS*	**2.1**	**3.8**	**2.9**	**3.3**	**1.8**	**2.2**	0.2	0.2	apoptosis promoter
*IRAK1*	-0.7	-0.4	-0.4	-0.3	0.1	-0.1	**1.6**	0.1	apoptosis inhibitor
*MALT1*	na	na	na	na	na	na	**1.5**	0.8	apoptosis inhibitor
*SERPINB2*	**1.1**	**2.5**	0.2	**1.7**	0.6	**1.3**	nd	nd	inhibits TNF-alpha-induced apoptosis
*TNFRSF6B*	na	na	na	na	na	na	**-2.7**	**-2.0**	apoptosis inhibitor

^a^Log_2 _ratios from 13 K cDNA microarrays (DNR) and ^b ^log_2 _ratios from 8.5 K oligonucleotide microarrays (Affymetrix).

na = gene not on array; nd = not detected; genes scored as up-regulated (log_2 _ratio ≥ 1) or down-regulated (log_2 _ratio ≤ -1) are in bold print.

^c ^Information from NCBI Entrez Gene.

Cell cycle alterations at 8 and 24 hours after drug addition were reflected in altered expression patterns of genes involved in cell cycle progression in the treated HCT116 cell lines compared to untreated controls. Cell cycle- and growth-regulatory genes such as *AREG, CCND3 *and *CDKN1A *were up-regulated in all 5-FU-treated cell lines compared to untreated controls at either 8 or 24 hours or both, whereas down-regulation of *CCNB1 *was detected in ContinD cells only. *CCNC*, *CCNG1*, and *CDC25B *were down-regulated in parental and ContinB cells, whereas *RAN *was down-regulated in ContinB cells (Table 1). There was good correlation between *CDKN1A *expression measured by real-time RT-PCR and that measured using the 13 K microarrays (r = 0.7, p < 0.05). CDKN1A protein levels at 8 hours were 1.9, 2.6, and 1.7-fold higher in the treated parental, ContinB, and ContinD cells respectively relative to their untreated controls, whereas the corresponding levels were 2.8, 3.8 and 2.0-fold higher at 24 hours (Figure 8c). MYC was also induced in response to 5-FU treatment in all HCT116 cell lines; protein levels at 8 hours were 2.3, 2.4, and 3.2 fold higher in the treated parental, ContinB and ContinD cells, respectively compared to their untreated controls, and at 24 hours, these levels had increased 2.9, 2.4 and 4.8-fold compared to their respective controls (Figure 8d). MYC protein levels did not correlate with transcript levels, since the *MYC *transcript was down-regulated in parental and ContinB cell lines (slight down-regulation in ContinD cells) (Table 1). The cell cycle-regulatory genes *CDC6*, *CDCA5*, *PDAP1*, *PDXP*, *PVT1*, and *RARRES2 *were altered in the parental HCT116 cell line but not in either of the 5-FU-resistant cell lines in response to short-term drug treatment. The S-phase regulatory gene *PPP2CB *was up-regulated in all cell lines, whereas *RRM2 *was up-regulated in parental and ContinB cells. *MCM3 *was down-regulated in all cell lines, consistent with reduction or cessation of replication activity. In agreement with the reduced entry into mitosis, spindle-checkpoint and mitosis-regulatory genes such as *BUB1*, *BUB1B*, *NEK4*, *PLK *and *STK6 *were all down-regulated at 8 and/or 24 hours in these cell lines in response to 5-FU.

Table 1 summarizes the expression levels of apoptosis-regulatory genes that were altered at 8 and 24 hours following drug addition in the HCT116 parental and resistant cell lines. The apoptosis-inhibiting genes *AVEN *and *SERPINB2 *were up-regulated at both 8 and 24 hours. The p53-regulated apoptosis-promoting gene *FAS *was up-regulated in each cell line, but lowest FAS levels were seen in ContinD cells. The apoptosis-promoting gene *BNIP3L *was down-regulated in all cell lines, while the apoptosis-promoting gene *CASP3 *was down-regulated in ContinB cells only.

Some of the genes whose expression levels had been altered as a consequence of resistance development were further altered in response to short-term 5-FU treatment (8 or 24 hrs.) (Table 1), e.g. *AREG*, *ATF3*, *BNIP3L*, *CCND3*, *CCNG1*, *CDKN1A*, *CHC1*, *GADD45A*, *MCM3*, *PLK*, and *STK6*. Interestingly, some genes that were initially down-regulated as a result of resistance development (*AREG*, *CDKN1A*, *GADD45A*) were up-regulated in response to short-term 5-FU treatment. The opposite was also true; some genes that were initially up-regulated as a result of resistance development (*CHC1*, *MCM3*, *PLK*, and *STK6*) were down-regulated in response to short-term drug treatment.

### Differences in 5-FU-induced gene expression in 5-FU-resistant cell lines

Having discussed genes specifically involved in DNA damage response, cell cycle and apoptosis regulation, we next focused on the genes that showed the largest differences in 5-FU-induced expression in the cell lines with different resistance levels (Table 2). A set of genes coding for guanine nucleotide-binding proteins (G proteins), which integrate signals between membrane receptors and downstream effector proteins, showed marked differential expression after 5-FU treatment in the 5-FU-resistant cell lines. *GNL3 *was only down-regulated in ContinD cells, while *GNL2 *was only down-regulated in ContinB cells. Neither of them were altered in the parental cell line in response to 5-FU. Other genes involved in nucleoside/nucleotide metabolism were also differentially expressed. *ATAD2 *was down-regulated in ContinD cells, whereas *CMPK*, *MATR3*, *PRPS2 *and *TNRC8 *were altered in ContinB cells. Genes involved in mRNA processing/transport (e.g. *EPRS*, *GNB2*, *STAU1*, *SYMPK*) were down-regulated in the parental cell line but not in the resistant cell lines. Other differentially-expressed genes were involved in regulation of cytoskeleton organization and cell adhesion: *KRT7*, *KRT8*, *KRT19*, all up-regulated in ContinB cells; *JUP*, up-regulated in ContinD cells and down-regulated in ContinB cells; *ACTG2*, *ARPC3*, *F2*, down-regulated in parental cells. Genes involved in amino acid metabolism (e.g. *AHCY*, *GSS*, *IVD*, *OAT*) were all down-regulated in ContinB cells. Signal transduction and transport genes such as *NCBP1*, *RAN*, *SNAPAP*, *TM9SF2 *were down-regulated in ContinB cells, whereas *CENTG3 *and *TAX1BP1 *were altered in ContinD cells. *VPS52 *was down-regulated in parental HCT116 cells. Some of the genes whose expression levels were altered in response to short-term 5-FU treatment had also been altered as a consequence of resistance development, e.g. *IVD *and *TAX1BP1*.

**Table 2:** Differential gene expression in parental HCT116 and 5-FU-resistant cell lines: additional affected regulatory pathways.

Gene symbol	par 8 hr 5 FU^a^	par 24 hr 5 FU^a^	ContinB 8 hr 5 FU ^a^	ContinB 24 hr 5 FU ^a^	ContinD 8 hr 5 FU ^a^	ContinB as a result of 5 FU	altered in ContinD as a result of resistance development ^b^	altered in ContinD as a result of resistance development ^b^	Regulatory pathway/function
									***Nucleoside/nucleotide metabolism and nucleotide binding***
*ADSS*	-0.1	**-1.4**	0.1	-0.6	-0.2	-0.3	0.8	0.6	GTP and nucleotide binding; nucleotide metabolism
*ATPIF1*	-0.2	-1.0	0.4	-0.1	0.0	**-1.5**	na	na	negative regulation of nucleoside metabolism
*ATAD2*	-0.7	-0.5	-0.6	-0.6	-0.3	**-1.2**	na	na	ATP and nucleotide binding; assembly/disassembly of protein complexes
*CMPK*	0.6	0.5	0.9	**1.2**	0.0	0.4	0.0	0.1	nucleobase, nucleoside, nucleotide and nucleic acid metabolism
*GNL2*	-0.2	0.3	**-1.9**	**-1.2**	-0.2	-0.2	0.2	0.1	nucleotide binding, protein binding, GTPase activity
*GNL3*	0.1	-0.2	-0.5	0.0	-0.4	**-1.7**	-0.5	-0.3	nucleotide binding, protein binding, GTPase activity
*MATR3*	-0.2	-0.8	-0.5	**-1.4**	-0.1	0.0	-0.6	-0.1	RNA binding; metal ion binding; nucleotide and nucleic acid binding
*MBNL1*	**-1.0**	**-1.8**	0.3	-0.3	-0.8	-0.4	na	na	nucleic acid binding; zinc ion binding
*PRPS2*	0.1	-0.3	**-1.6**	**-1.2**	0.0	-0.2	-0.2	-0.2	nucleoside metabolism; nucleotide biosynthesis
*TNRC8*	0.4	0.7	0.6	**1.3**	-0.2	-0.1	na	na	trinucleotide repeat containing 8 gene – function unknown
									
									***mRNA processing/transport***
*EPRS*	-0.3	**-1.5**	0.2	0.0	-0.7	-0.1	-0.9	-0.6	catalyzes aminoacylation of glutamic acid and proline tRNA species
*GNB2*	-0.5	**-2.2**	-0.3	-0.6	-0.1	0.3	0.1	-0.2	pre-mRNA processing and cytoskeleton assembly
*HMG20B*	-0.6	**-2.1**	-0.7	-0.4	-0.2	-0.2	na	na	transcription factor; tRNA ligase activity; ATP binding
*SRRM1*	-0.6	**-1.7**	-0.4	-0.2	-0.3	-0.4	0.8	0.1	mRNA processing
*STAU1*	-0.3	**-1.2**	0.3	0.0	-0.1	-0.4	0.4	-0.1	mRNA transport; intracellular transporter activity
*SYMPK*	**-1.9**	-0.9	-0.4	-0.8	-0.3	-0.3	0.3	-0.1	role in HSF1 modulation of Hsp70 mRNA polyadenylation
*THOC1*	-0.5	**-1.3**	-0.5	-0.7	-0.1	-0.4	-0.3	0.4	regulates transcriptional elongation; mRNA export from nucleus
									
									***Cytoskeleton/cell motility/cell adhesion***
*ACTG2*	-0.1	**-1.3**	0.0	-0.6	-0.1	-0.4	nd	nd	maintenance of the cytoskeleton; cell motility
*ARPC3*	0.0	**-1.2**	-0.2	-0.6	0.2	-0.2	0.4	0.5	regulation of actin cytoskeleton
*F2*	-0.4	**-1.2**	0.4	0.1	-0.1	-0.6	nd	nd	regulation of actin cytoskeleton; post-translational modification of proteins
*ILK*	-0.4	-0.3	0.0	0.7	0.1	**-1.1**	-0.2	-0.3	cell-matrix adhesion; integrin-mediated signaling pathway
*JUP*	-0.4	0.2	**-1.1**	-0.5	-0.1	**1.1**	-0.8	-0.3	cell adhesion
*KRT7*	0.1	0.4	0.8	**1.4**	0.3	0.4	na	na	cytoskeleton organization and biogenesis; cell communication
*KRT8*	0.3	0.1	**1.0**	**1.7**	0.3	0.6	na	na	cytoskeleton organization and biogenesis; cell communication
*KRT19*	0.3	0.7	**1.0**	**1.7**	-0.1	0.5	-0.3	-0.9	cytoskeleton organization and biogenesis; cell communication
*MAST1*	0.2	-0.7	**-1.4**	**-1.3**	0.1	0.2	na	na	cytoskeleton organization and biogenesis; protein amino acid phosphorylation
*TUBB*	0.0	-0.3	0.5	0.3	-0.2	**-1.3**	na	na	microtubule-based movement; protein polymerization
*TUBE1*	-0.5	-0.6	0.3	-0.3	-0.3	**-1.0**	na	na	microtubule-based movement; protein polymerization
									
									***Amino acid, protein, carbohydrate metabolism***
*AHCY*	0.7	0.9	**1.3**	**1.4**	0.2	0.3	0.2	-0.3	methionine, selenoamino acid metabolism
*GANAB*	-0.2	-0.5	**-1.4**	**-1.3**	0.1	-0.2	na	na	carbohydrate metabolism
*GCLC*	-0.5	-0.9	**-2.2**	**-1.6**	-0.2	-0.3	na	na	glutamate metabolism
*GSS*	-0.3	-0.3	**-1.8**	-0.8	0.0	0.4	-0.2	-0.6	glutamate and glutathione metabolism
*IVD*	-0.7	-0.2	**-1.6**	**-2.4**	-0.3	0.3	**1.0**	0.1	valine, leucine and isoleucine degradation
*MRP63*	0.7	0.6	**1.4**	**1.1**	0.2	0.0	na	na	protein synthesis within the mitochondrion
*OAT*	0.1	-0.4	**-1.9**	**-1.7**	-0.1	0.0	-0.5	0.1	arginine and proline metabolism; ornithine metabolism
									
									***Signal transduction/transport***
*CENTG3*	**-1.1**	0.2	-0.5	0.2	-0.6	**1.1**	na	na	regulation of GTPase activity; small GTPase mediated signal transduction
*ERP70*	-0.2	**-1.9**	-0.9	-0.4	-0.1	-0.3	na	na	electron transport and protein secretion
*KPNA3*	-0.8	**-1.2**	-0.1	-0.3	-0.3	-0.2	-0.1	-0.1	involved in nuclear transport system; intracellular protein transport
*MTCH1*	0.6	0.7	**1.2**	**1.4**	0.4	0.3	0.1	0.0	neuronal ion channel clustering; transport; regulation of signal transduction
*NCBP1*	-0.3	-0.6	**-1.1**	**-1.0**	0.0	-0.3	0.0	0.0	RNA splicing; mRNA nuclear export; mRNA processing; transport
*RAB8A*	-0.3	**-2.4**	-0.1	-0.7	0.1	-0.2	0.5	0.1	GTP/GDP-binding protein involved in protein transport
*RAN*	-0.6	-0.8	**-1.2**	**-1.6**	0.0	-0.3	na	na	RNA and protein export from nucleus; small GTPase mediated signal transduction; intracellular protein transport
*SLC16A1*	0.2	**-1.3**	0.5	-0.5	-0.1	-0.7	-0.1	-0.5	mevalonate and organic anion transporter activity
*SNAPAP*	-0.4	-0.9	-0.9	**-1.6**	-0.1	-0.2	na	na	exocytosis; intracellular protein transport; neurotransmitter secretion
*TAX1BP1*	-0.6	-0.1	0.6	0.6	0.2	**-1.1**	**-1.3**	-0.7	protein binding
*TAX1BP3*	-0.3	**1.6**	-0.1	0.0	0.3	-0.2	na	na	ATP binding; signal transduction; ion transport
*TM9SF2*	-0.4	-0.5	**-1.9**	**-1.6**	-0.1	-0.5	0.1	0.1	transport
*TPD52*	-0.4	-0.7	**-1.8**	**-1.5**	-0.3	-0.3	na	na	calcium ion binding; protein binding; protein homodimerization activity; secretion
*UFM1*	-0.9	-0.4	-0.6	**-1.5**	0.1	0.3	na	na	ubiquitin cycle – function unknown
*VPS52*	-0.6	**-1.2**	0.1	-0.5	0.1	-0.2	0.3	-0.1	involved in vesicle trafficking from endosomes to trans-Golgi network
									
									***Oxygen metabolism***
*COX5A*	0.8	0.4	**1.2**	**1.0**	0.2	0.1	-0.1	-0.1	oxidative phosphorylation
*COX7C*	0.6	0.3	0.3	**1.1**	0.3	0.2	na	na	oxidative phosphorylation
*MPV17*	-0.3	0.5	**-1.3**	0.5	-0.4	0.9	-0.1	-0.1	oxygen and reactive oxygen species metabolism
*SDHB*	-0.1	-0.7	-0.7	**-1.6**	0.1	-0.2	-0.1	-0.3	oxidative phosphorylation; oxidative decarboxylation of pyruvate and TCA cycle

^a ^Log_2 _ratios from 13 K cDNA microarrays (DNR) and ^b^log_2 _ratios from 8.5 K oligonucleotide microarrays (Affymetrix).

na = gene not on array; nd = not detected; genes scored as up-regulated (log_2 _ratio ≥ 1) or down-regulated (log_2 _ratio ≤ -1) are in bold print.

^c ^Information from NCBI Entrez Gene.

## Discussion

Cell cycle progression after DNA damage is regulated by checkpoint controls in the G_1 _or G_2 _phase of the cell cycle. Additionally, S phase progression is reduced, but not entirely halted, after DNA damage [11]. Arrest in G_1 _and G_2 _allows repair prior to replication and mitosis, respectively.

Failure to repair can result in apoptosis, mitotic catastrophe, or senescence [6]. In the present work, we wanted to elucidate potential differential responses to 8 and 24-hour 5-FU treatment in the HCT116 parental cell line and its 5-FU-resistant derivatives. We assessed several cellular phenotypes in an effort to clarify potential differences: levels of 5-FU uptake into DNA, cell cycle effects and apoptosis induction throughout treatment and recovery periods for each cell line. Each cell line incorporated 5-FU into DNA, but levels of incorporation were not significantly different between the cell lines at either 8 or 24 hours. 5-FU led to a G_1_(/S) arrest at 8 and 24 hours, consistent with the results of previous studies [7,12,13]. The G_1 _arrest was most pronounced in ContinD cells at 24 hours, whereas the S phase arrest was most pronounced in parental HCT116 cells at 24 hours. It also appeared that ContinD cells had a higher tendency to arrest in G_2_. Cell counts began to decrease immediately for parental and ContinB cells following 5-FU removal from the media, whereas this decrease was delayed by 24 hours for ContinD cells. Decreases in cell numbers following drug removal were consistent with cessation of mitotic activity at 24 hours and with subsequent high levels of apoptosis (for parental and ContinB cell lines) during recovery. The pattern of cell cycle progression during recovery demonstrated consistently that the smallest S phase fractions and the largest G_2_(/M) fractions were measured in the 5-FU-resistant cell lines.

The levels of apoptosis were dramatically lower in the ContinD cell line relative to the other two cell lines, a pattern that persisted throughout the recovery period. Since this cell line also experiences a dramatic cell loss (>95%, Figure 1), which is not the result of apoptosis, it may be that cell death in this cell line occurs via necrosis. In any event, this cell line had the fastest turnaround time, in that it recovered exponential growth within 10 days, compared to 20 days for the ContinB cell line and closer to 30 days for the parental cell line.

The G_1_(/S) arrest in these cell lines was accompanied by increases in p53 protein levels and induction of *CDKN1A *transcripts and CDKN1A, suggesting that the arrest could be p53-mediated. p53 is known to play a central role as a mediator of the DNA damage response/cell cycle arrest and in apoptosis induction [1,14,15]. There was little agreement between p53 protein levels and *TP53 *transcript levels, since the latter were either unchanged or down-regulated at 8 and 24 hours in each cell line. However, the mechanism of p53 protein activation is by protein stabilization (and phosphorylation) rather than by increased transcription [4], and since these cell lines have wild-type *TP53 *[10], and CDKN1A transcript and protein is induced after irradiation with ionizing radiation (unpublished results), the p53 response appears to be normal in the resistant cell lines as well as in the parental cell line. 5-FU treatment for 24 hours resulted in up-regulation of p53-target genes on DNA damage response/repair (*GADD45A*, *XPC *[16]*PCNA *[17], *TP53I3*, and *ATF3*), cell cycle-regulatory (*CDKN1A*), and apoptosis-regulatory pathways (*FAS*) in the parental and resistant cell lines. Differential down-regulation of cell-cycle regulatory genes known to be repressed by p53, e.g. *PLK, CCNB1, CCNB2 *and *TOP1 *[18] was also demonstrated in these cell lines. Successful detection of known p53-target genes by the microarrays used in the present work indicated that we had a good system for identifying p53-responsive genes. Apoptosis induction also appeared to be p53-mediated, as the p53-dependent apoptotic promoter *FAS *was up-regulated [19,20] in these cell lines in response to 5-FU treatment. Furthermore, induction of apoptosis is substantially reduced in these cell lines following knockdown of p53 (manuscript in preparation).

Alterations in gene expression levels on cell cycle-, apoptosis-, and DNA damage response-regulatory pathways in the present study provided little explanation for the differential resistance to 5-FU seen in the three cell lines, especially that seen in the ContinD cell line compared to the other two cell lines. Many of the same genes were altered in response to 5-FU in all three cell lines, with only small differences in expression levels measured. Additionally, some of the genes that were up-regulated in response to short-term drug treatment had originally been down-regulated as a consequence of resistance development [10], e.g. *CDKN1A*, *GADD45A*, and *AREG *(Table 1), underscoring the difficulty in elucidating their role in/contribution to an overall resistance phenotype and the intricacy of drug resistance generally. However, when we considered other cellular regulatory pathways that were affected in response to short-term drug treatment, we found that genes involved in nucleotide binding and nucleotide metabolism, mRNA processing, cytoskeletal organization, amino acid metabolism, signal transduction/transport, and oxygen metabolism were differentially altered in the three cell lines (Table 2). Some of the affected genes were altered in the parental cell line but not in the resistant cell lines (mRNA processing genes), or in one or both resistant cell lines but not in the parental cell line (amino acid and nucleotide metabolism genes). Such gene alterations may provide important information about pathways that are activated in response to 5-FU in cells that are already resistant to the drug, information which may have useful implications for the design and modification of current chemotherapeutic regimens.

## Conclusion

Our gene expression data suggest that altered regulation of nucleotide metabolism, amino acid metabolism, cytoskeleton organization, transport, and oxygen metabolism may underlie the differential resistance to 5-FU seen in these cell lines. Future work will involve RNA interference studies to assess the contributory roles and importance of some of the altered genes to 5-FU resistance.

## Methods

### Cell lines, culture conditions, and drug treatment

The HCT116 parental cell line (ATCC CCL247) and HCT116 ContinB and ContinD resistant derivatives (all wild-type *TP53 *cell lines) were cultured in RPMI medium with 10% heat-inactivated fetal calf serum, 2 mM glutamine, and 0.6% Pen-Strep at 37°C in a humidified atmosphere with 5% CO_2_. 5-FU was purchased from Amersham Biosciences, England. HCT116 parental and resistant cells were seeded out at a density of 1.0 × 10^5 ^cells per ml. in 6-well plates for cell cycle and apoptosis assays, in 25 cm^2 ^flasks for 5-FU incorporation measurements, and 75 cm^2 ^flasks for gene expression analyses. 770 μM 5-FU was added to the media 24 hours after seeding. Cells were harvested by trypsinization or scraping at 0, 8, and 24 hours after addition of 5-FU to the culture medium. Control wells received no 5-FU. For gene expression analyses, media was aspirated from exponentially-growing parental and resistant HCT116 cell cultures, at 0 hours, 8 hours and 24 hours following addition of 770 μM 5-FU. Control cell cultures (without 5-FU) were also harvested at the same timepoints. Monolayers were harvested by scraping and the cells frozen at -80°C until used for RNA extraction.

### Incorporation of 5-FU into DNA

HCT116 parental and resistant cells were seeded out at a density of 1.0 × 10^5 ^cells per ml. in 25 cm^2 ^flasks. Cells were exposed to 5-FU for 24 hours as for the other assays, except that the 5-FU solution contained 425 nM of [6-^3^H]5-FU (Moravek Biochemicals Inc., Brea, CA). Control wells received no 5-FU. Cells were harvested by scraping at 8 and 24 hours after addition of 5-FU to the culture medium, and frozen at -80°C until used for DNA extraction. DNA was extracted using standard phenol:chloroform:isopropyl alcohol procedures, precipitated with 7.5 M ammonium acetate and absolute ethanol, washed with absolute ethanol, air-dried and then dissolved in Tris-EDTA buffer, pH 8.0. DNA concentrations were measured using absorbance spectrometry.

DNA samples were mixed into scintillation fluid and measured in a Tri-carb Packard liquid scintillation counter (Packard Instrument Co, Meriden, CT). Results are expressed as the ratio of disintegrations per minute (dpm): μg DNA.

### Cell counts

Trypsinized cell suspensions were counted using a standard Trypan Blue viability assay. Cell counts were performed at 0, 8, and 24 hours following addition of 5-FU to the medium. For recovery assays, cell counts were also done at successive 24-hour intervals until the cells had regained exponential growth. After cell counting, the same cell suspensions were then fixed in 80% ethanol for subsequent cell cycle analyses.

### Cell cycle analyses and quantification of apoptosis

Trypsinized cell suspensions were fixed in 80% ethanol. The samples were then placed at -20°C until cell cycle analysis. Nuclei were isolated from fixed cell suspensions, stained with propidium iodide (50 μg/ml), and samples analyzed for DNA content using a FACSCalibur laser flow cytometer (Becton Dickinson Immunocytometry Systems, San Jose, CA). Pulse-processed fluorescence signals were used to exclude doublets and aggregates from analyses. Ten thousand events were acquired for each sample. Percentages of cells in the G_1_, S, and G_2_M phases of the cell cycle were quantified using WinCycle software (Phoenix Flow Systems, San Diego, CA). Quantification of 5-FU-induced apoptosis during treatment and recovery periods in each cell line was done using the sub-G_1 _peaks from the cell cycle analyses measured during these periods.

### Mitotic cell discrimination

Percentages of mitotic cells in control and 5-FU-treated cell cultures were determined using a flow cytometric method to discriminate mitotic cells as described previously [21]. Trypsinized cell suspensions were centrifuged at 1000 rpm for 5 minutes, washed once with PBS, and resuspended in 750 μl of a cooled detergent buffer (0.1% NP40, 6.5 mM Na_2_PO_4_, 1.5 mM KH_2_PO_4_, 2.7 mM KCl, 137 mM NaCl, 0.5 mM EDTA, pH7.2). After 5 min. on ice, the cells were fixed by adding 250 μl 4% formaldehyde to give a final concentration of 1%, mixed well, and allowed to fix for a minimum of 1 hr. on ice. Samples were then centrifuged at 1200 rpm for 5 minutes and the pellets resuspended in the detergent buffer containing 5 μg/ml propidium iodide and 100 μg/ml RNaseA. Samples were analyzed on a FACSCalibur laser flow cytometer and percentages of mitotic cells were measured using correlated DNA content/forward scatter distributions.

### Microarray hybridization and data analysis

RNA isolation, preparation of Cy3- and Cy5- fluorescently-labeled cDNA samples, and subsequent hybridization to 13 K microarrays were done as described previously [22]. Thirty micrograms total RNA of control and drug-treated cells were used for the Cy3- and Cy5-labeled samples, respectively. The 13 K cDNA microarrays were prepared at the Radiumhospital microarray core facility, and information about them can be found at their website [23]. Hybridized slides were scanned using a Scan Array 4000 laser scanner at 10 μm resolution (Packard BioChip Technologies, Billerica, MA). Spot and background intensities, and the standard deviations of these, were quantified using Quantarray software (Packard BioChip Technologies). Bad spots and regions with high unspecific binding of dye were manually flagged and excluded from the analysis. Background-subtracted intensities less than two times the standard deviation of the local background were assigned this value to avoid zero or negative values in the ratio calculations. Weak spots with background-subtracted intensity less than two times the standard deviation of the local background in both channels were excluded. Total intensity normalization of the data was performed [24]. Genes in the 5-FU-treated HCT116 cell lines that had two-fold expression level changes (signal log_2 _ratios ≥ 1 or ≤ -1) relative to corresponding untreated controls at 8 hours or 24 hours were scored as up-regulated or down-regulated respectively as a result of drug treatment. At the 8 hour timepoint following 5-FU addition, a total of 88, 99, and 10 genes were scored as altered in HCT116 parental, ContinB, and ContinD cells, respectively. At 24 hours, these numbers had increased to 218, 323, and 89 for the ratios) for the same cell lines. A text-tab-delimited file of all gene expression data (log_2 _ratios) for the 5-FU-treated parental and drug-resistant HCT116 cell lines (relative to their respective untreated controls) is available upon request.

Gene expression data was sifted using GenMapp version 2.0 (Gene MicroArray Pathway Profiler) software [25]. Use of this program facilitated an immediate and comparative overview of genes scored as up-regulated or down-regulated (signal log_2 _ratios ≥ 1 or ≤ -1, respectively) on specific pathways in response to 5-FU treatment in parental and 5-FU-resistant HCT116 cells. We focused on altered genes located on DNA damage stimulus response, cell cycle (general regulation, S phase and M phase regulation) pathways and apoptosis regulatory pathways.

### Real-time RT-PCR

Expression levels for 2 genes, *GADD45A *and *CDKN1A *were determined by real-time RT-PCR for HCT116 parental, ContinB, and ContinD treated and untreated control cells at all treatment timepoints using Taqman Gene Expression Assays (Applied Biosystems, Foster City, CA). 200 ng of total RNA was subjected to real time RT-PCR using an ABI PRISM Sequence Detection System following manufacturer protocols, in order to confirm the 13 K microarray results. Primers are available upon request. The 18S gene was used as an endogenous control for equal amounts of RNA used.

### Western analyses

Scraped cell suspensions including floating cells that had loosened from the monolayer during the course of 5-FU treatment were centrifuged and the pellets heated in standard Laemmli buffer containing PMSF. Protein concentrations were quantified (BioRad, Hercules, CA), and protein samples (15 μg) and Precision Protein molecular weight standards (6.5 μg, BioRad) were separated by SDS-PAGE (10% or 12% gels) and transferred to PVDF membranes (BioRad). Western blotting was performed using mouse monoclonals against human p21WAF1 and p53 (clones EA10 and Pab1801, respectively, Calbiochem, San Diego, CA), MYC (clone 6E10, Cambridge Research Biochemicals, USA), GADD45A (C-4, Santa Cruz Biotechnology, San Diego, CA) and actin (C-2, Santa Cruz Biotechnology – used as a loading control). An amplified alkaline phosphatase staining procedure (BioRad) was used to detect the separated proteins. Expression levels were quantified using UnScanIt gel software version 5.1 for Windows (Silk Scientific Inc., Orem, Utah).

## Authors' contributions

PMD conceived of and designed the study, carried out the 5-FU incorporation assays, mitotic cell discrimination analyses, apoptosis analyses, and gene expression analyses. DHS performed the microarray hybridizations. KLK was responsible for the cell culture work and performed the cell cycle analyses and Western analyses. TS supervised the microarray work and participated in the gene expression analyses. All authors read and approved the final manuscript.
